# ^1^H-NMR Urinary Metabolic Profile, A Promising Tool for the Management of Infants with Human Cytomegalovirus-Infection

**DOI:** 10.3390/metabo9120288

**Published:** 2019-11-25

**Authors:** Marie Antoinette Frick, Ignasi Barba, Marina Fenoy-Alejandre, Paula López-López, Fernando Baquero-Artigao, Paula Rodríguez-Molino, Antoni Noguera-Julian, Marta Nicolás-López, Asunción de la Fuente-Juárez, Maria Gemma Codina-Grau, Juliana Esperalba Esquerra, Ángeles Linde-Sillo, Pere Soler-Palacín

**Affiliations:** 1 Pediatric Infectious Diseases and Primary Immunodeficiencies Unit, Pediatrics Department, Children’s Hospital, Vall d’Hebron Barcelona Hospital Campus, 08035 Barcelona, Spain; 2 Red de Investigación Translacional en Infectología Pediátrica (RITIP), 28046 Madrid, Spain; 3 Cardiovascular Diseases, Vall d’Hebron Research Institute, Vall d’Hebron Barcelona Hospital Campus, 08035 Barcelona, Spain; 4 Centro de Investigación Biomédica en Red sobre Enfermedades Cardiovasculares (CIBER-CV), 28029 Madrid, Spain; 5 Pediatrics Infectious Diseases Unit, Pediatrics Department, Hospital University La Paz, 28046 Madrid, Spain; 6 Infectious Diseases and Systemic Inflammatory Response in Pediatrics, Infectious Diseases Unit, Pediatrics Department, Hospital Sant Joan de Déu, 08950 Barcelona, Spain; 7 Hospital Sant Joan de Déu Pediatric Research Institute, University of Barcelona, 08950 Barcelona, Spain; 8 CIBER de Epidemiología y Salud Pública, CIBERESP, 28029 Madrid, Spain; 9 Pediatrics Department, Hospital Terrassa, C/Torrebonica, 08227 Barcelona, Spain; 10 Pediatrics Department, Hospital Quirónsalud Barcelona, 08023 Barcelona, Spain; 11 Microbiology Department, Vall d’Hebron Barcelona Hospital Campus, 08035 Barcelona, Spain; 12 Neonatology Department, Vall d’Hebron Barcelona Hospital Campus, 08035 Barcelona, Spain

**Keywords:** human cytomegalovirus, congenital infection, pediatrics, metabolomics, ^1^H-NMR, metabolic profiling

## Abstract

Congenital human cytomegalovirus (HCMV) infection is the most common mother-to-child transmitted infection in the developed world. Certain aspects of its management remain a challenge. Urinary metabolic profiling is a promising tool for use in pediatric conditions. The aim of this study was to investigate the urinary metabolic profile in HCMV-infected infants and controls during acute care hospitalization. Urine samples were collected from 53 patients at five hospitals participating in the Spanish congenital HCMV registry. Thirty-one cases of HCMV infection and 22 uninfected controls were included. Proton nuclear magnetic resonance (^1^H-NMR) spectra were obtained using NOESYPR1D pulse sequence. The dataset underwent orthogonal projection on latent structures discriminant analysis to identify candidate variables affecting the urinary metabolome: HCMV infection, type of infection, sex, chronological age, gestational age, type of delivery, twins, and diet. Statistically significant discriminative models were obtained only for HCMV infection (*p* = 0.03) and chronological age (*p* < 0.01). No significant differences in the metabolomic profile were found between congenital and postnatal HCMV infection. When the HCMV-infected group was analyzed according to chronological age, a statistically significant model was obtained only in the neonatal group (*p* = 0.01), with the differentiating metabolites being betaine, glycine, alanine, and dimethylamine. Despite the considerable variation in urinary metabolic profiles in a real-life setting, clinical application of metabolomics to the study of HCMV infection seems feasible.

## 1. Introduction

Human cytomegalovirus (HCMV) is a ubiquitous viral agent that affects more than half the world’s population. The most severe clinical manifestations are seen in immunocompromised patients and in infants with prenatal mother-to-child HCMV transmission causing congenital HCMV infection (cHCMV).

cHCMV is associated with hearing loss, microcephaly, retinitis, vision loss, developmental and motor delay, seizures, etc. The reported prevalence of congenital HCMV infection (cHCMV) is 0.2% to 2% of all pregnancies, making cHCMV the most common congenital infection in the developed world [1,2]. HCMV infection can be asymptomatic or symptomatic at birth. Half the symptomatic and 13% of asymptomatic infected patients will develop significant long-term neurological or audiological impairment [1,3,4]. Antiviral treatment improves the audiological and neurodevelopmental outcomes in symptomatic patients [5,6]. When in utero diagnosis fails, newborns are diagnosed through viral isolation or detection of viral genetic material using polymerase chain reaction (PCR) testing in urine or saliva within the first two weeks of life. Unfortunately, the diagnosis of asymptomatic cases at birth, and particularly the identification of those who will develop long-term sequelae, is still a challenge. Identifying these infants is an area of strong clinical need. It would ensure that these babies receive prompt treatment and follow-up opportunities, to improve their clinical prognosis [7,8].

cHCMV must be differentiated from postnatal HCMV infection (pHCMV). pHCMV occurs when an infant acquires HCMV after birth through breast-feeding, contact with maternal secretions during labor, blood transfusions, or nosocomial transmission. It is usually asymptomatic, except in very-low-birth-weight premature infants. The prevalence of pHCMV in premature babies is approximately 6% to 22%, and 14% of these will have a severe disease (hepatitis, neutropenia, thrombocytopenia, sepsis-like syndrome, pneumonitis, or enterocolitis) [9,10]. Unlike cHCMV disease, pHCMV in the preterm infant does not seem to be associated with hearing loss or neurodevelopmental abnormalities long-term [9].

Differentiation between patients with cHCMV or pHCMV after the first three weeks of life can be complex, especially when the mother is immune to HCMV, no cHCMV stigma are detected, and neonatal dried blood spot PCR tests negative due to its limited sensitivity [11,12,13,14]. This scenario reflects the need for further research to improve the identification and management of cHCMV, in which the youngest “omic” science, metabolomics, could play a role [7].

Metabolomics, also referred to as the “new clinical biochemistry”, involves a systematic study of the complete set of low-molecular-weight metabolites present in a biofluid or tissue sample [15]. This process delivers a full description of the metabolic status in relation to genetic and epigenetic factors, pathological conditions, and external stimuli, and shows an unbiased, true picture of cells, tissues, or organisms even before full expression of a phenotype [3,7,8,16,17,18]. Metabolomics may take us closer to a more personalized type of medical care, in which the “right medicine is given to the right patient” [19,20,21]. However, although the field of metabolomics has greatly advanced in recent years, application of these techniques has not yet reached the clinical stage. This may be because technical and biological challenges remain to be solved; among them, the higher interindividual variability of patients in real-life practice due to comorbidities, differing treatments, and other confounders, than those in pre-clinical studies, where these factors are more controlled [22,23].

Urine is a particularly appropriate biofluid for the study of metabolomics in pediatric and especially, neonatal conditions, as it contains concentrations of numerous metabolites and can be collected in a noninvasive manner. Urine has been considered a “window” on the organism that can provide valuable information on the changes in human metabolism [3,16,24]. A number of perinatal and neonatal conditions, including HCMV infection, have been investigated using metabolomics in preclinical studies [3,16,25,26,27,28,29]. The use of this approach in neonatology is still in a pioneering phase, but it is believed that if more information about the neonatal metabolic status were available, the management of these patients could be improved and even individually tailored, thereby rendering benchtop research into bedside clinical practice [16,30]. Nonetheless, most studies to date have not addressed one of the main problems limiting the translation of “omics” to neonatology clinical practice, namely, the high interindividual variability of neonates with acute illness [31].

The aim of this study was to conduct an observational study in the acute hospital setting to improve biological knowledge of HCMV infection in infants. Particularly, to investigate the effects of HCMV infection and other factors on the urine metabolic profile of newborns and infants and to evaluate whether this approach could be feasible and useful in a hospital clinical setting. We have chosen proton nuclear magnetic resonance (^1^H-NMR) spectroscopy combined with multivariate statistical analysis due to its reproducibility and the fact that it is inherently quantitative [32].

## 2. Results

### 2.1. Patients

The study included 31 infants with HCMV infection (11 cHCMV and 20 pHCMV) and 22 uninfected controls admitted to the critical care units of participating hospitals for various conditions during the study period. It is important to note that the cases group had a number of conditions in addition to HCMV infection. Comorbidities in both cases and controls included prematurity, intrauterine growth restriction (IUGR), diaphragmatic hernia, congenital heart disease, esophageal atresia, cystic fibrosis, polymalformative syndrome, 22q11 deletion syndrome, and severe combined immunodeficiency. There were no differences between the groups except for the chronological age at urine collection, with the controls being younger than the cases (*p* = 0.0054). As the patients were in the acute setting their hydration status was controlled periodically. The clinical and epidemiologic data of the study patients are shown in Table 1.

### 2.2. Pattern Recognition

**Figure 1:**
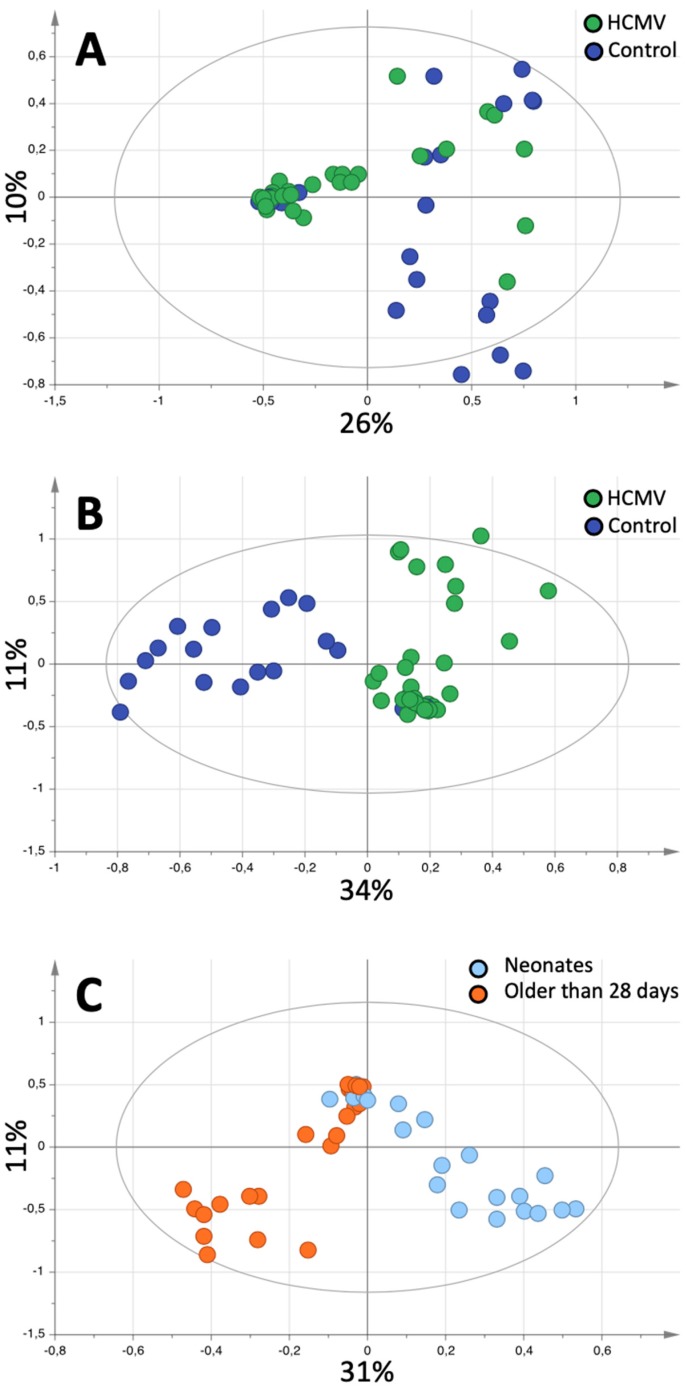
Pattern recognition analysis of Human Cytomegalovirus (HCMV) infection fingerprinting. (**A**) Score plot of the Principal Component Analysis (PCA) of the spectra, colored according to HCMV infection (green) or controls (blue). (**B**) Score plot of the Orthogonal Projections to Latent Structures Discriminant Analysis (OPLS-DA) model differentiating between controls and HCMV-infected patients. (**C**) Corresponds to the score plot of the OPLS-DA analysis differentiating between neonates (light blue) and infants older than 28 days of life (orange). Each dot corresponds to a spectrum/sample/patient.

In all cases, good quality ^1^H-NMR spectra were obtained from the participants’ urine samples. On visual inspection, the spectra obtained showed considerable interindividual variation (Figure S1). Principal component analyses (PCA) showed a tendency towards classification of infected versus uninfected patients in the first component (Figure 1A). None of the other variables of interest (type of infection [cHCMV, pHCMV], sex, chronological age, gestational age, corrected age, type of delivery, multiple birth, or diet) were associated with sample clustering. After application of Orthogonal Projections to Latent Structures Discriminant Analysis (OPLS-DA), we obtained statistically valid models able to identify HCMV infection (*p* = 0.03) (Figure 1B). It was also possible to distinguish between the spectra according to chronological age (*p* = 0.003) (Figure 1C). Table 2 shows the results of the various OPLS-DA models obtained when classifying the variables of interest. Of note, although it was possible to obtain a model to differentiate between cHCMV and pHCMV, it did not reach statistical significance according to CV-ANOVA (Figures S2 and S3).

The ^1^H-NMR peaks enabling differentiation of HCMV-infected patients from controls in the supervised model were found at 1.93, 3.04, 3.26, and 3.57 ppm (Figure 1B). Based on chemical shift, these peaks were tentatively assigned to acetate at 1.93, creatinine at 3.04, betaine at 3.26, and glycine at 3.57 ppm. The OPLS-DA model was able to identify infected patients according to chronological age (Figure 1C), infants older than 28 days showed a relative increase in the acetate peak, whereas neonates showed a relative increase in glycine and creatinine.

**Figure 2:**
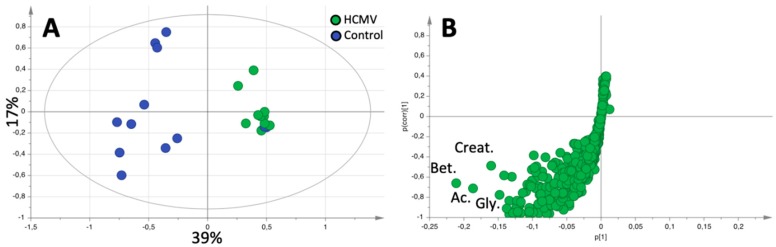
(**A**) Score plot corresponding to the OPLS-DA model differentiating between HCMV-infected (green) and control (blue) samples in infants younger than 28 days. (**B**) shows the s-plot of the models shown in (**A**) with the most relevant metabolites in the discrimination model highlighted. Creat, Creatinine; Bet, Betaine; Ac, Acetate; and Gly, Glycine.

The next step was to create OPLS-DA models to differentiate HCMV-infected patients from uninfected controls, taking into account the patients’ chronological age. We defined two groups according to age, neonates (younger than 28 days of life), and infants older than 28 days of life (up to 122 days). A statistically significant model able to differentiate between patients with HCMV infection and controls could only be obtained in neonates (R^2^X of 0.561, R^2^Y of 0.615, Q^2^ of 0.514; *p* = 0.012) (Figure 2A).

In total, 25 metabolites were identified and quantified from the urine spectra. The incidence of each of these varied considerably (Table 3). Only 5 metabolites were present in all the samples analyzed; a finding that further highlights the variation between samples.

Finally, we quantified and compared metabolite concentrations after dividing participants into four groups according to age at assessment and HCMV infection: neonate case, case older than 28 days, neonate control, and control older than 28 days. We found no differences in urinary metabolites between cases and controls in babies older than 28 days of life. In contrast, in neonates, betaine (*p* = 0.048), glycine (*p* = 0.015), alanine (*p* = 0.038), and dimethylamine (*p* = 0.048) concentrations were lower in the HCMV-infected group than in the control group (Table 4 and Table 5). There were no differences in creatinine content (*p* = 0.3) between the two groups; however, when metabolite concentration was normalized to creatinine content only glycine and alanine showed statistical differences (Tables S1 and S2).

## 3. Discussion

In this study, we used an ^1^H-NMR-based metabolomics approach to diagnose HCMV infection in urine of infants in a hospital acute care setting. The spectra obtained showed considerable interindividual variation that was consistent with the study patients’ wide variety of clinical conditions, which were representative of the populations admitted to the participating hospitals. Despite this elevated variability, it was possible to establish a metabolic fingerprint associated with HCMV infection that included increases in alanine, betaine, dimethylamine, and glycine. Of the detected and quantified metabolites, it is worth noting that two samples contained polyethylene glycol, the most likely origin for this compound is as an intravenous vehicle for pharmacological treatment.

The literature contains few reports describing the ^1^H-NMR urine spectra of newborns in a hospital setting. An early study by Trump et al. showed that spectra from healthy babies are similar to those of adults [33]. The background variation in the spectra we obtained was higher than the effects of HCMV infection reported previously in a much more controlled environment [7]. We could not attribute the spectral differences observed to sample collection; thus, the differences can be ascribed to the wide variety of clinical conditions of the patients included in the study [34].

Due to the considerable variation in the spectral patterns, we were unable to identify a particular trend associated with HCMV infection on visual inspection alone. This may be because urine spectra are difficult to fully understand, as they are based on a chemically complex biofluid [35]. However, PCA showed a tendency towards sample clustering, according to HCMV infection. This led to a statistically significant discriminant model that enabled differentiation between patients with HCMV infection and controls. The metabolites manifesting these differences were acetate, creatinine, betaine, and glycine. Of note, metabolite quantification validated the pattern recognition results except for acetate, which did not show significant differences between the groups.

Betaine, dimethylamine, and glycine were all found to be decreased in HCMV infected neonates when compared to controls. Dimethylamine and glycine can originate from the metabolic degradation of betaine. Furthermore, betaine is associated to one-carbon metabolism and may serve as a source of one-carbon units when other sources (folate, methionine) become limited [36]. One-carbon metabolism is relevant in health and diseases even more in the population under study in this work which consists mainly in premature babies [37]. Taken together, our data would suggest a reduction in one-carbon metabolism in the case of HCMV infected infants which could explain the cognitive impairment often seen in these babies.

Chronological age also generated a statistically significant model, in which the discriminating metabolites were acetate, creatinine, and glycine. Therefore, we conducted a further analysis using metabolite quantification and comparison of cases and controls according to their chronological age. In this analysis, neonatal HCMV-infected patients were differentiated from the control group by the following metabolites: betaine, glycine, alanine, and dimethylamine. However, the discrimination power was lost in infants older than 28 days. When the two types of infection were compared (cHCMV vs. pHCMV), a statistically significant model could not be obtained. As Trump et al. reported, the urinary metabolome in infants changes with age and other factors such as the diet, which may also add to the variability with age [13,34]; among the changes, it has been described that one-carbon metabolite levels tend to normalize within days after birth [38].

Previous experimental studies have shown that HCMV infection induces changes in the host metabolism by redirecting the host’s physiological functions to provide the virus with energy and precursor molecules for its own replication, thus affecting normal cellular metabolic balance and establishing a specific metabolic signature for the virus [8,39,40,41]. Hence, detection of HCMV infection by studying the urinary metabolic profile makes sense. However, the experimental models of viral infection may not fully describe the true metabolic processes occurring in the human body. This is one of the strengths of our study, as it is in vivo. Furthermore, it takes place in the acute hospital setting, where patients are admitted for various conditions, as was reflected by the spectra obtained. Nonetheless, we were able to find statistically significant differences.

Fanos et al. examined the effect of cHCMV infection on the urinary metabolome in 23 newborns whose mothers had HCMV infection during pregnancy and compared 12 cHCMV cases with 11 uninfected controls [7]. As the study population was not described in detail, a direct comparison with the patients in our study is not possible. The authors also used a supervised analysis to differentiate between cases and controls. The metabolites responsible for the difference were 3-hydroxybutyrate, 3-aminoisobutyrate, taurine, betaine, and scyllo-inositol, which were higher in the infected group, and myo-inositol, glycine, and ethanolamine, which were lower in the infected group compared to controls. Glycine and betaine are the only discriminant metabolites identified in both the present study and that of Fanos et al. The differences in trend between our work and that of Fanos et al. could be due to the population under study, while we work in the acute setting with other co-morbidities there is little data regarding clinical data in the work of Fanos et al. Also, the only information of sample collection is that it is in the first three weeks after birth and it is known that the metabolic profile changes within [38].

One of the advantages of working with metabolomics is that it is a hypothesis-generating discipline. The data obtained can lead to the discovery of new discriminant metabolites or metabolic pathways that had not been considered previously, and these can be complementary to other, previously detected, metabolites [22,42].

Reported data suggest that other sources of variation, including age, type of delivery, twins, gestational age, postnatal maturation, diet, and IUGR may have an impact on the neonatal urinary metabolome [13,25,27,43,44,45,46]. Using our dataset, we were unable to obtain statistically significant models for any of these factors, with the exception of age. This can be explained by the limited sample size, as the database contained only 8 IUGR patients and up to 5 different diets. The wide chronological age range at assessment may be the reason why no discriminant models were obtained for mode of delivery, gestational age, or multiple birth.

The limitations of this study include a relatively small sample size, especially regarding cHCMV. To mitigate the effect of this limitation, we evaluated OPLS-DA models using CV-ANOVA, a robust approach. Furthermore, urine metabolite quantification confirms the results obtained using pattern recognition. However, further studies with larger sample number should be carried out to support the findings. The high interindividual variation of the spectra/metabolites obtained could be an advantage as it allowed observation of several pathologies at the same time, but also a limitation because the analysis involved a complex data set [24,42].

In this study we have tried to answer a relatively simple question, if it is possible to diagnose HCMV infection though the urine metabolic profile and some aspects of the pathophysiology of HCMV infection remain unanswered. For example, we do not know if the metabolic changes observed are due to the virus infection or the symptoms. HCMV is able to replicate in the kidney, thus it is likely that kidney effects of HCMV infection are responsible for the differences in urine metabolome; animal experiments should be performed to confirm this point, but they are beyond the scope of our work.

Also, it is difficult to differentiate between the effects of HCMV infection and the co-morbidities; for this, a direct comparison between healthy babies and HMCV infected ones could be done, but it would lack clinical relevance.

In conclusion, metabolomics may be a promising approach to better understand the effects of HCMV infection on metabolism and identify metabolic profiles associated with the diagnosis, severity, and prognosis of this condition [47]. This could lead us to a more tailored management strategy for infected patients, based on early prediction of outcome, particularly interesting in asymptomatic patients, and to better diagnostic techniques to differentiate between cHCMV and pHCMV when urine cannot be collected in the first two weeks of life.

The present work aimed at improving biochemical knowledge of HCMV infection in infants. HCMV infection is a clinically relevant condition in a small population of infants in the acute setting; while the background variation is high due to different clinical conditions the possibility of an early diagnosis of HCMV infection in this population is clinically relevant. Thus, while our results are promising, this study should be considered a proof of concept and larger multicenter studies will be required in order to fully characterize this group of patients.

## 4. Materials and Methods

### 4.1. Patients

The study included urine samples from 53 infants hospitalized from April 2016 to July 2018. Forty-six patients were recruited at the tertiary level neonatal unit of Vall d’Hebron Barcelona Campus Hospital, equipped with 25 intensive care cots, 20 intermediate care cots, and 24 special care cots. The remaining seven patients were recruited from four Spanish hospitals that actively participate in the Spanish national congenital HCMV cohort registry (REDICCMV): La Paz University Hospital (Madrid), Sant Joan de Déu University Hospital (Esplugues de Llobregat), Terrassa Hospital (Terrassa), and Quirón Hospital (Barcelona).

### 4.2. Definitions

Patients were classified as non-infected controls (*n* = 22) when urine HCMV PCR tested negative, and as cases (*n* = 31) when urine HCMV PCR tested positive. HCMV-infected patients were further classified as having cHCMV when urine HCMV PCR was positive in the first two weeks of life. pHCMV was diagnosed when the urine HCMV PCR was positive after the first two weeks of life in a patient with a previously negative result. The study was approved by the ethics committee of clinical research of Vall d’Hebron Barcelona campus hospital (PR(AG)179/2016) and by the other participating hospitals. Informed consent was obtained from parents before enrolment in the study. All methods were performed in accordance with the relevant guidelines and regulations. The patients’ clinical charts were reviewed, and sex, chronological age, gestational age, corrected age (defined as number of weeks born before 40 weeks of gestation subtracted from chronological age), weight, type of delivery, multiple birth, diet, and comorbidities were recorded.

### 4.3. Samples

Urine samples were collected using urine collection bags in term and late preterm infants, and cotton wool balls in the most premature infants. Urine samples were collected at different times of the day for HCMV infection screening. Once urine had been collected and HCMV PCR had been performed at the microbiology department, the remaining urine was frozen and stored at −80 °C until analysis. The urinary CMV viral load was analyzed as follows: DNA was extracted from the urine specimen using the Nuclisens EasyMag system (BioMérieux, Mary-l’Étoile, France) and CMV viral load was determined using the RealStar CMV PCR Kit 1.2. (Altona diagnostics, Hamburg, Germany). In August 2018, before ^1^H-NMR analysis on the metabolomic platform of the Vall d’Hebron research institute, samples were thawed and homogenized with a vortex. A 450 μL amount of urine was centrifuged at 12,000× *g* at 4 °C during 10 min to precipitate any solid debris. A 400 μL amount of supernatant was mixed with 200 μL of phosphate buffer solution (0.14 M NaCl, 0.0027 M KCl, and 0.10 M Phosphate pH 7.4 (uncorrected reading)) prepared with D_2_O and containing TSP at a final concentration of 1 mM. The mixture was further centrifuged at 12,000× *g* at 4 °C for 10 min, and 550 μL of supernatant was transferred to a 5 mm NMR tube.

### 4.4. NMR Spectroscopy

One-dimensional spectra were acquired at 30 °C on a 9.4T vertical bore magnet interfaced to a Bruker Avance 400 console (Bruker, Madrid, Spain). For each spectrum, 128 scans were acquired with a NOESYPR1D pulse sequence with a mixing time of 100 milliseconds. All spectra were phased and baseline-corrected, and metabolites were identified and quantified using the Chenomx Profiler 8.0 software (Chenomx Inc., Edmonton, Canada) [48] by comparing the areas of the peaks of interest to that of the internal standard TSP. Metabolite quantification was measured independently by two researchers in order to ensure consistency.

### 4.5. Data Analysis

For metabolic profiling pattern recognition, each spectrum was divided into 1000 bins of equal width (0.01 ppm). After removing the water peak, the resulting digitized full spectrum was normalized to a total area of 1 and fed into SIMCA software (Umetrics, Umea, Sweden) for further processing. Inherent variation within the dataset was analyzed using principal component analysis (PCA). To assess the capacity of the ^1^H-NMR spectra to differentiate between classes, orthogonal projection on latent structures discriminant analysis (OPLS-DA), a supervised form of classification, was carried out. In this approach, Fisher linear discriminant analysis was used to find linear combinations of the variables (HCMV infection, sex, chronological age, gestational age, corrected age, type of delivery, multiple birth, diet, and comorbidities). The models were evaluated with misclassification tables and the help of ANOVA based on cross-validated predictive residuals (CV-ANOVA). OPLS-DA models were considered significant when the CV-ANOVA was <0.05 [7,49,50,51,52].

## Figures and Tables

**Table 1:** Clinical and epidemiological data of the patients included in the study.

Patient Characteristics	Cases (%)			Controls (%)	*p* *
**Number of patients**	**cHCMV** **11 (35.5)**	**pHCMV** **20 (64.5)**	**Total** **31 (58.5)**	**22 (41.5)**	
**Sex:** Male	5 (45.5)	11 (55)	16 (51.6)	11 (50)	ns
Female	6 (54.5)	9 (45)	15 (48.4)	11 (50)	
**Chronological age range (median), days**	0–37 (1)	35–122 (63)	0–122 (56)	0–115 (3)	0.006
**Chronological age:** ≤28 days of life	10 (90.9)	0 (0)	10 (32.3)	16 (72.7)	0.0054
>28 days of life	1 (9.1)	20 (100)	21 (67.7)	6 (27.3)	
**Gestational age range, weeks**	34–41	24^3/7^–41^5/7^	24^3/7^–41^5/7^	27–40^4/7^	ns
**Corrected age^, weeks:** Term	6 (54.5)	14 (70)	20 (64.5)	11 (50)	ns
Preterm	5 (45.5)	6 (30)	11 (35.5)	11 (50)	
**Birth weight, grams, range (median)**	1475–4000 (2455)	500–3840 (1035)	500–4000 (1475)	870–3955 (1710)	ns
**Type of delivery:** Cesarean section	7 (63.6)	16 (80)	23 (74.2)	15 (68.2)	ns
Vaginal delivery	4 (36.4)	4 (20)	8 (25.8)	7 (31.8)	
**Multiple birth**	3 (27.3)	7 (35)	10 (32.3)	5 (22.7)	ns
**Diet:** Breastfed	5 (45.4)	10 (50)	15 (48.3)	13 (59.1)	ns
Formula milk	2 (18.2)	6 (30)	8 (25.8)	7 (31.8)	
Mixed lactation	3 (27.3)	3 (15)	6 (19.4)	-	
Fasting	1 (9.1)	1 (5)	2 (64.5)	2 (9.1)	
**Supplement to main diet:** Intravenous serum	4 (36.4)	1 (5)	5 (16.1)	-	ns
Parenteral nutrition	-	1 (5)	1 (32.2)	7 (31.8)	
Fortification of breast milk	-	6 (30)	6 (19.4)	2 (9.1)	
**Other comorbidities:** Intrauterine growth restriction	3(27.3)	4(20)	7 (22.6)	1 (4.5)	ns
Congenital heart disease	-	1 (4.5)	1 (3.2)	2 (9.1)	
Diaphragmatic hernia	1 (9.1)	-	1 (3.2)	-	
Esophageal atresia	-	1(4.5)	1 (3.2)	-	
Cystic fibrosis	-	1(4.5)	1 (3.2)	-	
Polymalformative syndrome	-	-	-	1(4.5)	
22q11 deletion syndrome	-	1(4.5)	1 (3.2)	-	
Severe combined immunodeficiency	-	-	-	1(4.5)	

^ Corrected age is defined as number of weeks born before 40 weeks of gestation subtracted from chronological age. * Continuous variables were evaluated using t-test and categorical variables using chi-square; groups were considered different when *p* < 0.05.

**Table 2:** Summary of the OPLS-DA models created to differentiate between the groups of interest. An OPLS-DA model is obtained when samples are classified better than randomly. Those with CV-ANOVA < 0.05 were considered statistically significant.

Model	R^2^X	R^2^Y	Q^2^	*p* (CV-ANOVA)
HCMV infection vs. control	0.436	0.581	0.293	**0.03**
cHCMV vs. pHCMV	0.345	0.255	0.0847	0.499
Sex	0.237	0.378	0.0693	0.509
Chronological age	0.350	0.269	0.14	**0.003**
Gestational age	No Fit			
Corrected age	No Fit			
Type of delivery	No Fit			
Multiple birth	No Fit			
Diet	No Fit			

**Table 3:** Metabolites identified and quantified in the neonates’ urine samples. The percentage of samples in which the metabolite was detected is shown.

Metabolites	Concentration, mM (mean ± SD)	Incidence
1-Methylnicotinamide	0.346 ± 0.063	69%
Oxoglutarate	0.469 ± 0.099	40%
Acetate	1.451 ± 0.496	88%
Alanine	0.255 ± 0.049	79%
Betaine	0.949 ± 0.128	100%
Cadaverine	0.078	2%
Carnitine	0.542 ± 0.123	31%
Choline	0.122 ± 0.123	6%
Citrate	1.327 ± 0.240	62%
Creatine	0.377 ± 0.008	6%
Creatinine	1.323 ± 0.240	100%
Dimethylamine	0.271 ± 0.044	100%
Fumarate	0.045 ± 0.014	38%
Gallate	1.370 ± 0.923	4%
Glycine	1.280 ± 0.181	100%
Hippurate	0.151 ± 0.034	23%
Histidine	0.142 ± 0.024	48%
Lactate	0.404 ± 0.070	44%
N.N-Dimethylglycine	0.141 ± 0.033	98%
Propylene glycol	0.395 ± 0.129	8%
Succinate	0.340 ± 0.140	100%
Taurine	0.656 ± 0.114	40%
Threonine	0.051	2%
Tyrosine	0.045 ± 0.008	4%
myo-Inositol	1.523 ± 0.245	60%

**Table 4:** Urine metabolite concentrations mM (mean +/- SD) in neonates with and without HCMV infection.

Metabolites	Cases	Controls	*p*
**Acetate**	0.306 ± 0.624	0.606 ± 1.275	0.571
**Alanine**	0.052 ± 0.023	0.157 ± 0.147	0.038
**Betaine**	0.225 ± 0.070	0.619 ± 0.585	0.048
**Creatinine**	0.443 ± 0.389	1.081 ± 1.423	0.186
**Dimethylamine**	0.058 ± 0.046	0.226 ± 0.248	0.048
**Glycine**	0.300 ± 0.150	0.978 ± 0.790	0.015
**Succinate**	0.061 ± 0.117	0.094 ± 0.092	0.473

**Table 5:** Urine metabolite concentrations mM (mean +/- SD) in patients older than 28 days of life with and without HCMV infection.

Metabolites	Cases	Controls	*p*
**Acetate**	0.459 ± 0.935	0.909 ± 1.913	0.302
**Alanine**	0.078 ± 0.034	0.236 ± 0.220	0.456
**Betaine**	0.338 ± 0.105	0.929 ± 0.878	0.535
**Creatinine**	0.665 ± 0.583	1.622 ± 2.135	0.764
**Dimethylamine**	0.086 ± 0.069	0.339 ± 0.372	0.637
**Glycine**	0.450 ± 0.225	1.468 ± 1.185	0.781
**Succinate**	0.091 ± 0.176	0.140 ± 0.138	0.420

## Supplementary Materials

The following are available online at https://www.mdpi.com/2218-1989/9/12/288/s1, Figure S1: ^1^H-NMR spectra of three randomly selected urine samples to show considerable interindividual variation, Figure S2: Score plots corresponding to the OPLS-DA models shown in Table 2. Figure S3: Score plots corresponding to the PCA model with samples identified according to the hospital of origin, Table S1: Urine metabolites in neonates with and without HCMV infection, Table S2: Urine metabolites in patients older than 28 days of life with and without HCMV infection.
