# Supplementary material for: 1H-NMR Urinary Metabolic Profile, A Promising Tool for the Management of Infants with Human Cytomegalovirus-Infection
Source: Metabolites. 2019 Nov 25;9(12):288. doi: 10.3390/metabo9120288 (PMC6949898; doi:10.3390/metabo9120288)
Supplement: Supplementary file 1 [file metabolites-09-00288-s001.pdf]

**Supplementary Materials**

**Figures**

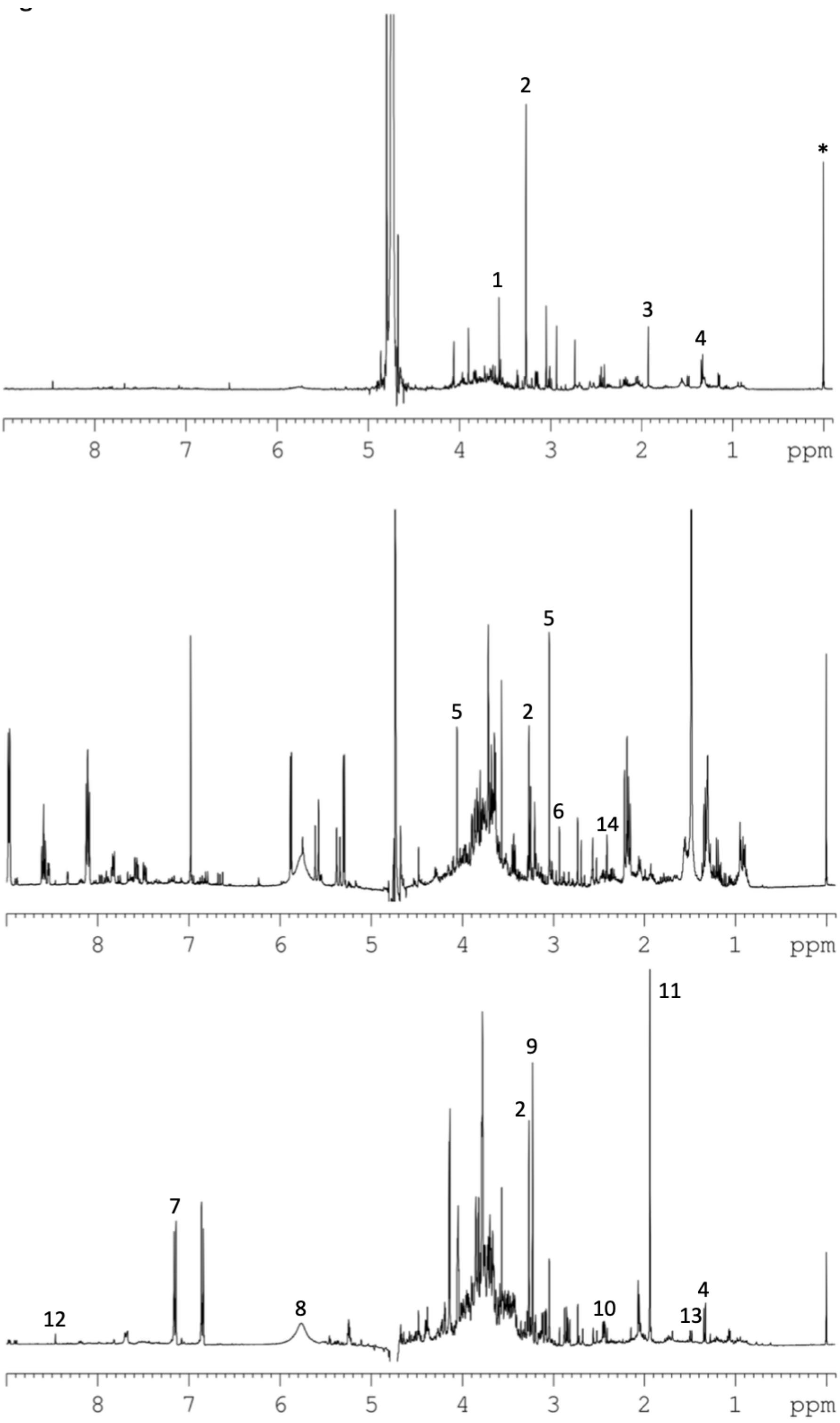


**Figure S1**. $^{1}$H NMR spectra of three randomly selected urine samples to show considerable interindividual variation. Tent[illegible]ssignations are: 1: glycine, 2: betaine, 3: acetate, 4: lactate, 5: creatinine, 6: chc[illegible] 7: tyrosine, 8: urea, 9: carnitine, 10: succinate, 11: citrate *TSP added as concentration and chemical shift reference.

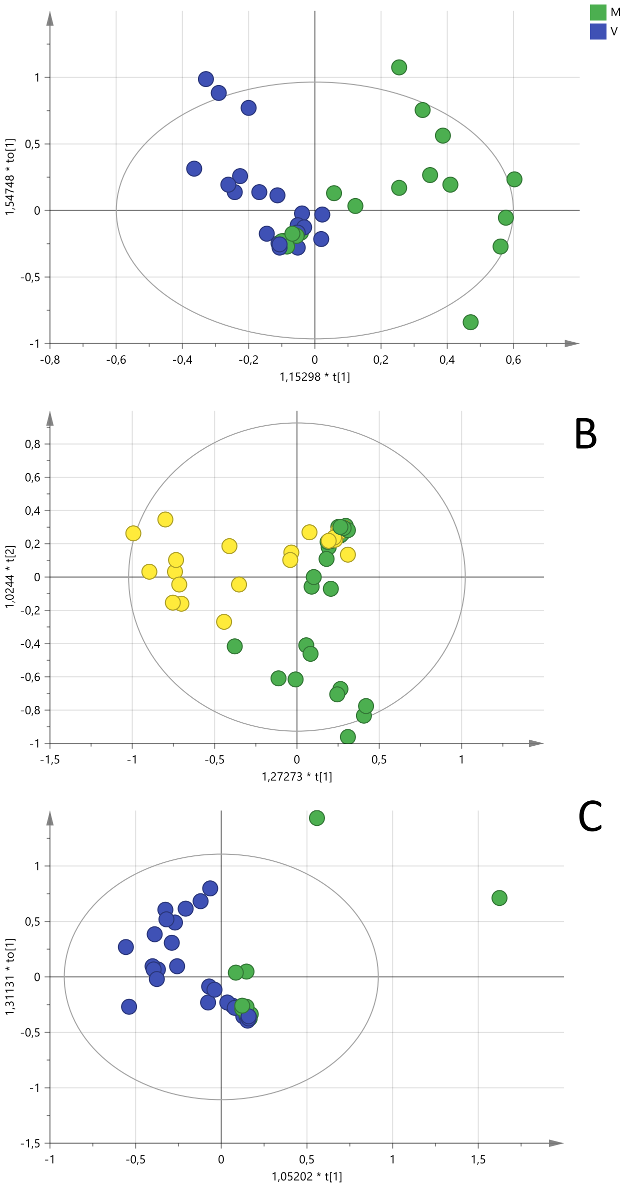


**Figure S2.** Score plots corresponding to the OPLS-DA models shown in Table 2. Only the one corresponding to chronological age was statistically significant according to CV-ANOVA. **(A)** OPLS-DA Score plot corresponding to boys (blue) and girls (green). **(B)** OPLS-DA Score plot corresponding to chronological age neonates (yellow) and infants older than 28 days (green). **(C)** OPLS-DA Score plot corresponding pHCMV (blue) and cHCMV(green).

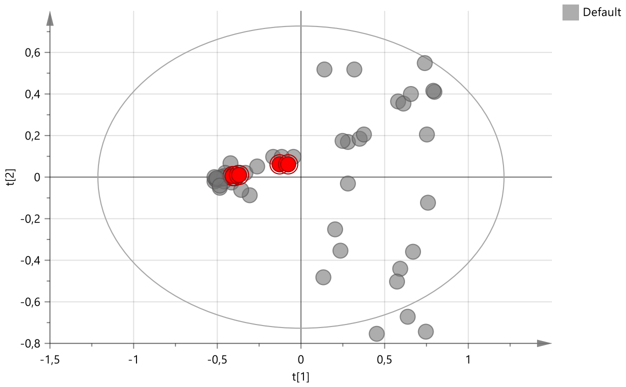


**Figure S3.** Score plots corresponding to the PCA model with samples identified according to the hospital of origin. Grey markings are samples from Hospital Vall d’Hebron, red markings other hospitals.

## Tables

**Table S1.** Urine metabolites in neonates with and without HCMV infection. Mmol of metabolite/mmol of total Creatinine. Data expressed as mean ± standard deviation.

| | HCMV | Control | p |
|---|---|---|---|
| Acetate | 1.11 ± 2.52 | 1.05 ± 2.09 | ns |
| Alanine | 0.14 ± 0.06 | 0.26 ± 0.15 | 0.031 |
| Betaine | 0.84 ± 0.62 | 0.77 ± 0.58 | ns |
| Dimethylamine | 0.16 ± 0.08 | 0.23 ± 0.21 | ns |
| Glycine | 0.76 ± 0.52 | 1.37 ± 0.68 | 0.030 |
| Succinate | 0.22 ± 0.47 | 0.13 ± 0.11 | ns |

**Table S2.** Urine metabolites in patients older than 28 days of life with and without HCMV infection. Mmol of metabolite/mmol of total Creatinine. Data expressed as mean ± standard deviation.

| | HCMV | Control | p |
|---|---|---|---|
| Acetate | 2.90 ± 4.04 | 0.83 ± 1.20 | ns |
| Alanine | 0.40 ± 0.38 | 0.26 ± 0.14 | ns |
| Betaine | 1.29 ± 0.72 | 0.87 ± 0.34 | ns |
| Dimethylamine | 0.28 ± 0.10 | 0.25 ± 0.11 | ns |
| Glycine | 1.41 ± 1.01 | 1.23 ± 0.80 | ns |
| Succinate | 0.66 ± 1.06 | 0.15 ± 0.14 | ns |
